# Telemonitoring for COVID-19 positive pregnant women; feasibility and user experience of SAFE@home Corona: prospective pilot study

**DOI:** 10.1186/s12884-022-04878-7

**Published:** 2022-07-11

**Authors:** Shinta L. Moes, Martine Depmann, Titia A. Lely, Mireille N. Bekker

**Affiliations:** Department of Obstetrics and Gynaecology, Wilhelmina Children’s Hospital, University Medical Centre Utrecht, KE.04.123.1, Lundlaan 6, 3584 EA Utrecht, The Netherlands

**Keywords:** COVID-19, Pregnancy, Telemonitoring, Digital health, Homemonitoring, SARS-CoV-2

## Abstract

**Background:**

COVID-19 has catalysed digital innovations enabling remote healthcare. Pregnant women are at increased risk for severe course of COVID-19 infection. Also, the pandemic has a negative emotional impact on pregnant women as they worry about their own health and the health of their unborn child. We developed a telemonitoring platform called SAFE@home-corona consisting of a pulse oximeter and an app with symptom checklist. The aim of this study was to examine the feasibility, defined by compliance to the platform and by monitoring the course of COVID-19, patient satisfaction and user experience of a telemonitoring platform in COVID-19 positive pregnant women in the Netherlands.

**Methods:**

We conducted a prospective pilot study among Dutch-speaking COVID-19 symptomatic pregnant women. Women were asked to monitor their oxygen-saturation with a pulse oximeter and COVID-related complaints with an in-app questionnaire daily. Oxygen-saturation and complaints were monitored by the Medical Management Centre with triage protocol. COVID-19, pregnancy, and user experience data were collected. To assess feasibility, compliance of daily self-monitoring and compliance of all intended measurements were calculated. Severity of COVID-19 was assessed via the platform and medical record. Patient satisfaction and user experience were measured through a self-developed questionnaire.

**Results:**

Twenty-eight women were eligible of which 27 (93.1%) completed the study. Compliance of daily measurement and all intended measurements was high with 98.9 and 93.9%, respectively. Six women were hospitalized, of whom one to the intensive care unit. Overall, women indicated high satisfaction scores, varying from 8 to 10/10. Women were more concerned for the health of their unborn child or family then for themselves (66.7%). They stated that the platform offered reassurance. Patients would highly recommend the platform to pregnant peers during COVID infection.

**Conclusions:**

This pilot study demonstrated feasibility of the SAFE@home-corona platform for self-monitoring COVID-19 course in pregnant women. Patients were satisfied, it offered reassurance, women would recommend use to peers. Upscaling the platform is needed to draw conclusions from the early signalling abilities and to keep evaluating patient satisfaction. The platform has great potential for self-monitoring of COVID-19 and possibly other pulmonary infections in pregnant women.

**Supplementary Information:**

The online version contains supplementary material available at 10.1186/s12884-022-04878-7.

## Background

Since the discovery of the novel SARS-Cov-2 virus (COVID-19), it has rapidly spread around the globe. Consequently, the large number of COVID-19 patients resulted in the downscaling of regular health care worldwide. This reduction of regular health care has catalysed digital health innovations during the current pandemic, enhancing the stimulation of remote health care [1]. In the Netherlands, 9620 cases of COVID-19 pregnant women were registered by the Netherlands Surveillance System (NethOSS) since the beginning of the COVID-19 pandemic in the first quartile of 2020 until November 2021 [2].

The pandemic has a negative emotional impact on pregnant women, they worry about their own health, the health of their unborn child and the inability to access reliable information [3]. Also, higher levels of depression and anxiety have been reported in pregnant women during the COVID-19 pandemic [4, 5]. Moreover, pregnant women are at increased risk for a severe course of COVID-19 infection [6–8].

In order to potentially recognize a severe course of COVID-19 in pregnant women early, close monitoring might be indicated. Close in-hospital monitoring of COVID-19 patients however is a strain on the hospital personnel and resources, since personal protective equipment and isolation facilities are required and it is over-treatment for most pregnant women, since only few of them will suffer from a severe COVID-19 infection. Therefore, it could be useful to monitor the course of COVID-19 related complaints in pregnant women from their homes. Considering the negative emotional impact of COVID-19 on pregnant women, at-home monitoring might provide them with much needed information and support.

Our research group has previously developed SAFE@home, a platform to telemonitor blood pressure and related complaints in pregnant women at high risk for hypertensive disorders [9]. By modification of the existing SAFE@home platform we developed a new platform: SAFE@home-corona, consisting of a tele-monitoring app combined with a pulse oximeter. This platform offers a method to monitor the complaints of COVID-19 positive pregnant women at home through an in- app questionnaire and to monitor their oxygen saturation and heart rate levels through the pulse oximeter. In this pilot study we evaluated the feasibility of this platform to monitor the course of COVID-19 in pregnant women and we evaluated the patient satisfaction and user experience of the platform.

## Methods

### Study design

We conducted a single-centre prospective observational pilot study in the University Medical Centre Utrecht (UMCU), which was approved by the local Medical Research Ethics Committee (METC 20–422-C). This study was performed in accordance with the Good Clinical Practice Guidelines and the Declaration of Helsinki. In the period from November 2020 until November 2021, women under care at the UMCU obstetric department were eligible to participate if they were pregnant at the time of a positive COVID-19 PCR test, either performed during hospital admission or by the Public Health Service. The women had to be Dutch speaking since the application language was in Dutch. There were no further exclusion criteria. Random sampling was used since all patients within our hospital that met inclusion criteria could participate in the study. Sample size for this study was 28 pregnant women.

To obtain information on the course of the COVID-19 infection, patients were asked to monitor their complaints and oxygen (O_2_) levels with the iHealth Air (by iHealthLabs Europe, Paris), an automatic pulse oximeter, and use Luscii (by Focuscura, The Netherlands), a smartphone application with an integrated questionnaire. In the questionnaire, women scored the severity of shortness of breath, coughing, rhinitis, sore throat and anosmia daily. Thresholds were predefined and set in the app and are shown in Supplementary Table 1 of Additional file 1. Thresholds were set for shortness of breath (> 3 points) indicating presence of severe shortness of breath in which case a patient could only walk a short distance at her own pace, temperature (> 38.4 degrees Celsius) indicating fever from 38.5 degrees Celsius onwards, coughing (> 4 point) indicating coughing that is almost constantly present, O_2_-saturation (< 96%) in line with a deviating sign on the national early warning score (NEWS) and heart rate (> 110 beats per minute) indicating 2-point tachycardia on the NEWS [10]. Furthermore, not filling out the questionnaire would give an alarm. In case a threshold was reached, a notification was sent to the web-portal of the hospital. Incoming alarms were checked by Master students of Medicine working in the Medical Management Centre (MMC) of the UMCU. The students checked the incoming alerts and handled them according to a predefined protocol. If, according to protocol it was deemed necessary, the supervising obstetric medical professional was consulted how to manage a certain case. If the complaints were under the threshold values for a minimum of 48 hours, patients were discharged from tele-monitoring.

Compliance of home self-monitoring was defined in a dual manner. The first was compliance of daily home self-monitoring defined as the number of days that measurements were filled in by the patients, divided by the total number of days patients were participating in the platform, multiplied by 100%. The second was compliance of all intended measurements defined as the total number of performed measurements divided by total number of intended measurements, multiplied by 100%.

To obtain information on patient satisfaction and user experience of the platform, a questionnaire consisting of 35 questions was developed by the local research team (See Supplementary Table 2 of Additional file 2). There was no time during the pandemic to validate the questionnaire on reliability before implementation, since there was a desperate need to reduce the pressure on the obstetric health care system in the UMCU by launching the home monitoring platform for the COVID pregnant patients. Items concerning patient satisfaction and user experience of the platform were constructed within the following domains: appropriateness (items 1–6, 13 and 14), acceptability (items 7, 8–12, 15–18, 21 and 22) and sustainability (item 23) as defined by Proctor et al. [11] Furthermore questions were created to collect user experience data on: feelings about health and the COVID infection (items 24–29), recommendation to peers (items 19, 20), information provision regarding COVID and pregnancy (items 30, 31) and experience with the MMC (items 32–34). Patients answered the questions on a 0 (totally disagree) to 10 (totally agree) Likert scale except for the open questions (items 2, 18, 24–26, 31, 33, 34 and 35).

### Data collection and analysis

From patients medical records and the Luscii platform information on duration and severity of COVID-19 related complaints and cases of hospital admissions due to COVID-19 were collected. Furthermore, data on pregnancy characteristics and outcome were collected. Body mass index (BMI) was calculated using preconception weight. After discharge from home monitoring, the researcher contacted women via telephone to conduct the questionnaire. All data was collected per patient in a case report file (CRF). Data analysis was performed with IBM SPSS Statistics, version 26. All CRFs were exported into the SPSS database. Descriptive statistics were used to calculate median and interquartile ranges (IQR) for baseline characteristics, COVID-19 and pregnancy outcomes. For the different items of the questionnaire descriptive statistics were also used for calculation of median scores and IQR.

## Results

During the study period, 28 pregnant women were identified as eligible to monitor their COVID-19 related complaints at home due to a positive PCR. No women refused to participate. Baseline characteristics of the 28 women participating in this study are depicted in Table 1.

**Table 1:** Baseline characteristics

Characteristic	***N*** = 28
Age, years (IQR^a^)	32 (29–36)
Nulliparity, n (%)	9 (32.1)
Body mass index in kg/m^2^ (IQR)	26.2 (22.5–30.9)
**Education, n (%)**	
Primary education	3 (10.3)
Secondary education	8 (27.6)
Post secondary education	11 (37.9)
University	6 (20.7)
**Ethnicity, n (%)**	
Caucasian	20 (71.4)
Middle-Eastern, North-African	8 (28.6)
**Smoking, n (%)**	
Currently not,	21 (75.0)
History of,	7 (25.0)
**Medication**	
Yes (%)	10 (35.7)
Immunosupressive, n	1
Antihypertensive, n	2
Anticoagulant, n	3
Hormones, n	2
Antidiabetics, n	2
**Care level at beginning of pregnancy n (%)**	
Primary	9 (32.1)
Secondary	12 (42.9)
Tertiary	7 (25.0)
GA^b^ on date of positive COVID-19 PCR^c^ test in weeks+days (IQR)	26^+ 6^ (20^+ 0^–32^+ 4^)

^a^*IQR* interquartile range

^b^*GA* gestational age

^c^*PCR* Polymerase Chain Reaction

The participants in this study had a median age of 32 years (IQR 29–36). The median BMI was 26.2 kg/m^2^ (IQR 22.5–30.9). 71.4% was of Caucasian ethnicity, 28.6% of Middle-Eastern or North-African ethnicity. Seven women (25.0%) had a history of smoking and 1 of them was smoking at time of inclusion. Regarding medication use, 10 patients (35.7%) were already using medication for an underlying condition. The median gestational age (GA) at the time of the COVID-19 infection was 26 weeks + 6 days.

One of the participants in this study discontinued self-monitoring because of few COVID-19 related complaints. We therefore excluded her from analysis. She did not have any adverse COVID-19 or pregnancy outcomes. Three women did not take part in the questionnaire section. In one woman, reason for this refusal was not specified, one woman found it too time consuming and one woman could not be reached despite repeated attempts.

With patients sending in any measurements on 183 days out of a total of 185 days, compliance of daily home self-monitoring was 98.9%. A total of 1389 measurements was sent in of the 1480 possible intended measurements, resulting in a compliance of all intended measurements of 93.9%.

The monitoring of complaints and O_2_-saturation via the platform lasted for a median duration of 6 days (IQR 4–7). The most common complaints of the participants were coughing (92.6%) and rhinitis (81.5%) (Table 2).

**Table 2:** Characteristics SAFE@home-corona platform

Characteristics	***N*** = 27
Days in the platform, n (IQR^a^)	6 (4–7)
Number of days past after positive PCR before start using app, n (IQR)	3 (2–5)
Number of logins (IQR)	6 (4–7)
**Compliance of**	
Daily home self-monitoring	98.9%
All intented measurements	93.9%
Fill in time in minutes n (IQR)	3 (2–5)
**Vital parameters measured**	
Lowest oxygen saturation in %, median (IQR)	96 (93.8–96.6)
Highest heart rate in beats per minute, median (IQR)	94 (85–106)
Highest temperature in degrees Celcius, median (IQR)	37.2 (36.5–37.8)
**Complaints related to COVID-19, n (%)**	
Shortness of breath	20 (74.1)
Coughing	25 (92.6)
Sore throat	15 (55.6)
Rhinitis	22 (81.5)
Loss of smell or taste	15 (55.6)
≥ 1 time(s) contact with MMC^b^, n (%)	15 (55.6)
≥ 1 time(s) contact with gynaecologist-in-training/supervisor	7 (25.9)

^a^*IQR* interquartile range

^b^*MMC* Medical Management Centre

Six women were admitted to the hospital, of which five to the obstetric ward and one to the intensive care unit (ICU). Four women were treated with anticoagulant, oxygen and corticosteroid therapy (Table 3). All recovered without noteworthy remaining symptoms and one birth was induced on maternal indication due to COVID-19 symptoms.

**Table 3:** Characteristics of COVID-19 infection (*n* = 27)

Characteristics	***N*** = 27
Admission indication: COVID-19, n (%)	6 (22.2)
Admission to obstetric ward, n (%)	6 (22.2)
Admission to intensive care unit, n (%)	1 (3.7)
Anticoagulant, oxygen and corticosteriod therapy, n (%)	4 (14.8)
Fully vaccinated at time of infection, n (%)	4 (14.8)

Twenty out of the twenty-seven women already gave birth, of which 12 spontaneous, one through vacuum extraction and seven through caesarean section (CS), of which four were unplanned/emergency CSs. One emergency CS was a premature delivery. This CS was performed on maternal indication since it was necessary to ventilate in prone position because of severe COVID-19 pneumonia. Two CSs were due to first stage arrest and the last one due to second stage arrest. The median GA at birth was 38 weeks + 5 days (38^+ 0^–41^+ 1^). The median birthweight was 3041 g (IQR 2825–3762). One stillbirth occurred of a fetus with a known lethal DNA repair disorder, this fetus was also small for gestational age. There were no unexpected cases of neonatal death. Three neonates were admitted to the NICU, the indications were prematurity, dysmaturity and respiratory problems due to meconium aspiration. Two of the neonates were PCR tested for COVID-19, both test results were negative.

Overall, women indicated high satisfaction scores for acceptability and appropriateness, usefulness varying from 8 to 10 out of 10. In particular, women were satisfied with the daily assessment of their O_2_-saturation level (with a score of 10 out of 10 [IQR 8–10]) and would highly recommend the platform to other pregnant women (with a score of 10 out of 10 [IQR 8–10]). Regarding user experience, more than half of the women expressed to be more concerned for the health of their unborn child, partner or family members then for themselves (66.7%). They stated that the monitoring platform offered reassurance during COVID-19 infection (Table 4). The most common feelings mentioned when hearing they were tested positive for COVID-19 were fear or insecurity (58.3%).

**Table 4:** Patient satisfaction and user experience

Characteristics	***N*** = 24
**Patient satisfaction** (appropriateness and acceptability)	**Median (IQR**^**a**^**)**
Utility and usefulness	
Q1. Usefullness app and saturation meter in general	8.0 (8.0–9.0)
Usefullness app and saturation meter:	
Q3. For course of corona infection	8.0 (8.0–9.0)
Q4. To obtain insight in my level of oxygen saturation	9.0 (8.0–9.0)
Q5. To experience more control over my health	8.0 (7.3–9.0)
Q6. To feel safer during my corona infection	8.0 (8.0–10.0)
Clarity of instructions	
Q8. Use of the saturation meter	9.5 (8.0–10.0)
Q9. Use of the application	9.0 (8.0–10.0)
Q10. The placement of any remarks	6.0 (5.0–8.0)
Q11. The to be received reminders	8.0 (8.0–10.0)
Q15. When to contact health care provider	8.0 (8.0–9.0)
Q16. Who to contact with health related issues	8.0 (8.0–9.0)
Q17.Who to contact with technical issues	7.0 (5.0–8.0)
O_2_- saturation mesurement experience	10.0 (9.0–10.0)
**Added value of SAFE@home-corona** (user experience)	
Q22. Safety or reassurance	9.0 (8.0–10.0)
**Contact with MMC**^**c**^**, Q34. (*n*** **= 15)** (user experience)	8.5 (7.8–10.0)
**Recommendation score** (user experience)	
Q19. Other COVID-19 patients	9.5 (8.0–10.0)
Q20. Other pregnant women	10.0 (8.0–10.0)

^a^*IQR* Interquartile Range

^b^*Q* Question

^c^*MMC* Medical Management Centre

## Discussion

In this pilot study we evaluated the feasibility of the SAFE@home-corona telemonitoring platform to monitor the course of COVID-19 related symptoms in pregnant women at home and calculated the compliance of home self-monitoring. Furthermore, we evaluated patient satisfaction and user experience of the platform.

During the study period four women were identified as potentially having a severe course of COVID-19 infection and were therefore admitted to the hospital. One of these women was admitted to the ICU. Patient satisfaction and user experience of the platform were highly valued amongst the participants. Compliance with daily self-monitoring was high.

In a time where regular check-ups were minimized due to the pandemic and more concern for health of mother and unborn child has arisen [3], SAFE@home-corona contributed to a more safe and reassured feeling for these patients.

In this pilot study the daily handling of incoming alarms from the SAFE@home-corona platform was redirected from the obstetric health care professional to the Medical Management Centre (MMC). The obstetric health care professional was only contacted by the Master students of Medicine working in the MMC if, according to protocol, this was deemed necessary. Moreover, when using the platform, there was only need for contact in case of a passed threshold, creating an alarm at the end of the MMC. This was clearly communicated to the patient during the instructions. These instructions, combined with the handling of the alarms by the MMC, lowered the burden for the obstetric healthcare professionals by reducing the number of phone calls or contact moments with included women. Naturally, in case of emergency, patients were instructed to call the emergency line of the birth centre or the emergency room for safety reasons.

Due to the small sample size of this feasibility study we cannot make a firm statement on the potential of the SAFE@home-corona platform to properly identify a severe course of COVID-19. However, four women were identified as possible severe COVID-19 infection using the platform thresholds. One of these women was admitted to the Intensive Care Unit. This indicates that on a case level it was possible to early contact patients with worsening symptoms and admit them to the hospital when thought necessary. Also, at-home monitoring through this platform might result in a reduction of healthcare costs through a decrease in hospital visits and possibly shorter hospital admission duration for pregnant women with COVID-19, which could. Ideally, we would like to ensure early admission of more severe COVID-19 patients so we could possibly prevent disease progression with early therapy. Whether oxygenation measurements in combination with daily complaint registration is useful for early admission and cost reduction remains to be investigated. When comparing the clinical parameters used in our questionnaire and the correlation to a severe course of COVID-19 to the existing literature, several studies found a strong association between hypoxemia and a worse clinical outcome in patients infected with COVID-19 [12–14]. Xie et al. found that an oxygen saturation of > 90.5% predicted survival of COVID-19 patients with a sensitivity of 84.6% and specificity of 97.2% [12]. Another study found an adjusted hazard ratio (aHR) of 1.36 for mortality in COVID patients with a saturation < 94% [15]. Compared to non-pregnant women of the reproductive age, pregnant women were less likely to report symptoms of dyspnoea, cough or fever. Also, fever in COVID patients has been harder to correlate to a worse clinical outcome, one study found that among hospitalized patients of an academic health care system in New York, only 30% presented with confirmed fever, defined as a temperature of ≥38 °C [16]. In our application the alarm for temperature was set at ≥38.5 °C, however the median highest temperature measured was far below that with 37.2 °C. For tachycardia or coughing we did not find significant predictive values regarding a severe course of COVID. It is important to emphasise that during the development of our platform there were no reliable cut-off points for clinical parameters to predict COVID course severity yet. However we chose safe boundaries with expert opinion and in accordance to existing local protocol, regarding oxygen-saturation the alarm was set from 96% downwards [17]. Oxygen therapy was started at saturation of < 94%, creating a safe window in which the patients’ other symptoms or complaints could be assessed [18].

Previous research has already shown the potential of tele-monitoring in obstetrics to improve patient empowerment and autonomy [19]. In COVID-19 patients, the use of several tele-monitoring initiatives have been proven to be safe and to reduce pressure on hospitals [20, 21]. The use of our tool for pregnant women, a population with higher risk of a more severe course of COVID [22, 23], has been rated with high patient satisfaction for both measuring O_2_-saturation and the use of the in-app questionnaire on symptoms. Women that used the app would also highly recommend the app to other pregnant women and patients with a COVID-19 infection in general. To our knowledge there has been one other study that investigated remote monitoring in pregnant women with COVID-19 related symptoms. McCabe et al. implemented a remote COVID-19 symptom monitoring program that used a text-based program that was linked to the electronic patient record [24]. Next to pregnant patients they included postpartum patients and patients with COVID-19 symptoms, whereas a positive SARS-CoV-2 PCR-test could have been performed but was not necessarily required. Patients were daily asked if they were feeling better or worse than the day before, there was no standard symptom checklist they daily had to answer as in our platform. Only in case of deterioration, difficulty with breathing was assessed. Different from our study, no saturation meter at home was available to the patient to objectify shortness of breath complaints. Similar to our results, McCabe et al. concluded that the implementation of a remote COVID-19 symptom monitoring system was easily adopted with high patient engagement.

There are a few strengths to our study. First, our platform is to our knowledge a pioneer in Europe regarding the home-monitoring of COVID-symptoms in pregnant women. Also, the combination with the Medical Management Centre is an innovative manner to save valuable time of obstetric health care professionals. Lastly, with the fast rise of digital health solutions in the last years and with the expectation that this transition will proceed in the future, it is important to continue to assess willingness of patients to use certain platforms and to evaluate the user-experience in feasibility studies such as this one [19, 25]. Through this manner, we have a better chance to ensure only workable and patient-valued digital platforms further develop into potential standard care. A limitation to our study is the small sample size. However, considering the purpose of this study was to asses feasibility of the SAFE@home-corona platform, we think the number of participants was sufficient. Furthermore, the questionnaire that was used has not been validated or tested on reproducibility since time was of the essence during the pandemic.

Upscaling of the SAFE@home-corona platform by including more women is needed to draw conclusions for the early signalling abilities of the platform and to keep evaluating patient satisfaction. This is important in light of the possibility of new COVID waves due to circulation of variants of SARS-CoV-2 [26]. Apart from COVID-19, this platform could have the potential to be used more widely for the monitoring of complaints of influenza or other severe viral lung infections and/or pulmonary disorders in pregnant women in the future and would therefore be interesting to investigate and test its feasibility.

In conclusion, this pilot study demonstrated that, in the setting of birth care at the UMCU, SAFE@home-corona is a feasible home tele-monitoring platform that is highly ranked in patient satisfaction and user experience and recommended to peers. It offers reassurance to pregnant women concerned for their own health and the health of their unborn child. Our platform has great potential for home self-monitoring of COVID-19 and other pulmonary infections in pregnant women.

## Supplementary Information

**Additional file 1: Table S1.** Threshold values of the SAFE@home-corona platform.

**Additional file 2: Table S2.** Questionnaire on patient satisfaction and user experience.

## Data Availability

The datasets used and/or analyzed during the current study are available from the corresponding author on reasonable request.

## Abbreviations

BMI: Body mass index
COVID-19: Coronavirus disease 2019
CS: Caesarean section
GA: Gestational age
ICU: Intensive care unit
IQR: Interquartile range
MMC: Medical Management Centre
NICU: Neonatal intensive care unit
UMCU: University medical centre Utrecht
