# Supplementary material for: Telemonitoring for COVID-19 positive pregnant women; feasibility and user experience of SAFE@home Corona: prospective pilot study
Source: BMC Pregnancy Childbirth. 2022 Jul 11;22:556. doi: 10.1186/s12884-022-04878-7 (PMC9272876; doi:10.1186/s12884-022-04878-7)
Supplement: Supplementary file 1 — Additional file 1: Table S1. Threshold values of the SAFE@home-corona platform. [file 12884_2022_4878_MOESM1_ESM.docx]

## Additional file 1. Threshold values of the SAFE@home-corona platform

##

Table S1. Threshold values of the SAFE@home-corona platform

| **Shortness of breath** | **Coughing** |
| --- | --- |
| 0 = none | 0 = none |
| 1 = very light | 1 = very light: a few coughs per day |
| 2 = light: after walking the stairs | 2 = light: every our or so |
| 3 = quite severe: need to adjust pace | 3 = quite severe: annoyingly present |
| **4 = severe: for example walking short distance at own pace** | **4 = very severe: coughing almost all of the time** |
| **5 = very severe: not yet at rust** | **5 = extremely severe** |
| **6 = extremly severe: at rest** |  |
| O_2_-saturation **< 96 %** | |
| Temperature **> 38.4 °C** | |
| Heart rate **> 110 beats per minute** | |
| General: **no measurement sent** | |
| Predefined values that were set in the application are shown. Shortness of breath and coughing had to be scored on a scale of 1 out of 6 and 1 out of 5, respectively. For O_2_-saturation, temperature and heart rate cut-off points were set. In case a patient did not sent any measurements there an alarm was generated. Threshold values that generated an alarm are displayed in bold. | |
