# Supplementary material for: Telemonitoring for COVID-19 positive pregnant women; feasibility and user experience of SAFE@home Corona: prospective pilot study
Source: BMC Pregnancy Childbirth. 2022 Jul 11;22:556. doi: 10.1186/s12884-022-04878-7 (PMC9272876; doi:10.1186/s12884-022-04878-7)
Supplement: Supplementary file 2 — Additional file 2: Table S2. Questionnaire on patient satisfaction and user experience. [file 12884_2022_4878_MOESM2_ESM.docx]

## Additional file 2. Questionnaire on patient satisfaction and user experience

Table S2. Questionnaire on patient satisfaction and user experience

| Questions and corresponding explanations | | Domain |
| --- | --- | --- |
| **Explanation: The following statements are about usefulness and experience** regarding the app and the saturation meter**: you can answer the question with a number between 0 and 10,**  **zero being you totally disagree and ten being you totally agree with the statement.** | |  |
| 1 | I thought the use of the Luscii app and the saturation meter were useful | Appropriateness |
| 2 | Why did you think so? | Appropriateness |
|  | The Luscii application and the accompanying saturation meter are useful: |  |
| 3 | for the course of my corona infection. | Appropriateness |
| 4 | to obtain insight in my level of oxygen saturation. | Appropriateness |
| 5 | to experience more control over my health. | Appropriateness |
| 6 | to feel safer during my corona infection. | Appropriateness |
| Explanation: The following question is about the **use of the saturation meter**: You can answer the question with a number between 0 and 10, zero being very annoying and ten being very pleasant. | |  |
| 7 | How did you experience the measurement of your oxygen level? | Acceptability |
| Explanation: The following statements are about the **use of the app and the saturation meter**: you can answer the question with a number between 0 and 10, 0 being you totally disagree and ten being you totally agree with the statement. | |  |
|  | The instruction that was given on (… a-d … ) was clear and understandable | Acceptability |
| 8 | a. the use of the saturation meter | Acceptability |
| 9 | b. the use of the application | Acceptability |
| 10 | c. the placement of any remarks | Acceptability |
| 11 | d. the to be received reminders | Acceptability |
| 12 | It is clear when I have to perform the measurements. | Acceptability |
| 13 | I am capable to perform the agreed measurements at home with the saturation meter and to submit the results. | Appropriateness |
| 14 | It is easy to look back at the course of my symptoms in the application. | Appropriateness |
|  | It is clear: |  |
| 15 | when I need to contact my healthcare provider. | Acceptability |
| 16 | who I need to contact in case of health related issues. | Acceptability |
| 17 | who I need to contact in case of technical problem. | Acceptability |
| 18 | How much time, in minutes, do you spend on the daily measurements? | Acceptability |
| Explanation: The following statements are about the **experience and recommendations** regarding the platform: you can answer the question with a number between 0 and 10, zero being you totally disagree and ten being you totally agree with the statement. | |  |
| 19 | I would recommend the platform to other patients to monitor their corona complaints. | User-experience |
| 20 | I would recommend the platform to other pregnant women to monitor their corona complaints. | User-experience |
| 21 | I trust the healthcare provider reviews my measurements and intervenes when necessary. | Acceptability |
| 22 | Because of the platform I feel safer. | Acceptability |
| 23 | I would like to use the platform again in case of a future corona infection. | Sustainability |
| **Feelings or experience during COVID infection** | |  |
| 24 | What went on in your mind when you heard you had corona? | User-experience |
| 25 | What was the reason for that? | User-experience |
| 26 | Would your reaction have been different if you were not pregnant? | User-experience |
| Explanation: The following statements are about the **perception after the positive corona test**: you can answer the questions with a number between 0 and 10, 0 being you totally disagree and ten being you totally agree. | |  |
| 27 | I was concerned about my own health after the positive test / start of the symptoms. | User-experience |
| 28 | I was concerned about the health of my unborn child after the positive test / start of the symptoms. | User-experience |
| 29 | I was concerned about the health of my partner/family after the positive test / start of the symptoms. | User-experience |
| 30 | I knew where I could go with my questions on COVID and pregnancy. | User-experience |
| 31 | If you do not agree, how can we improve this? | User-experience |
| **Medical Management Centre** | |  |
| 32 | Did the hospital contact you in response to your measurements? | User-experience |
| 33 | If yes, how many times? | User-experience |
| Explanation: The following question is about the **contact moments with the hospital**: you can answer the question with a number between 0 and 10, 0 being very annoying and ten being very pleasant. | |  |
| 34 | How did you experience these contact moments? | User-experience |
| 35 | Why did you experience it in that way? | User-experience |
